# Trends in the Prevalence and Mortality of Cervical Cancer in the Kyrgyz Republic

**DOI:** 10.7759/cureus.57863

**Published:** 2024-04-08

**Authors:** Abdrakhman Vaninov, Dzhyldyz Ibraimova, Aizhamal Sharshenbaeva, Altynai Zhumabekova, Olga Bolbachan, Bakyt Toktogaziev, Umetaly Sayakov, Yethindra Vityala, Tugolbai Tagaev, Fatima Dzhumabaeva

**Affiliations:** 1 Department of Special Surgical Disciplines, International Higher School of Medicine, Bishkek, KGZ; 2 Department of Public Health and Health Care, Kyrgyz-Russian Slavic University, Bishkek, KGZ; 3 Department of Oncology, I.K. Akhunbaev Kyrgyz State Medical Academy, Bishkek, KGZ; 4 Department of Obstetrics and Gynecology, City Maternity Hospital No. 2, Bishkek, KGZ; 5 Department of Faculty Surgery, I.K. Akhunbaev Kyrgyz State Medical Academy, Bishkek, KGZ; 6 Department of Pathology, International Higher School of Medicine, Bishkek, KGZ; 7 Department of Hospital Internal Medicine, I.K. Akhunbaev Kyrgyz State Medical Academy, Bishkek, KGZ

**Keywords:** early detection, socioeconomic status, screening programs, human papillomavirus, prevention, cervical cancer

## Abstract

Background: Cervical cancer represents a significant health concern globally and is the fourth most common cancer among women, leading to substantial morbidity and mortality. The primary cause is persistent infection with high-risk human papillomavirus (HPV) types. Despite advancements in prevention, screening, diagnosis, and treatment, disparities in cervical cancer outcomes persist due to variations in screening accessibility and socioeconomic factors. This study focuses on women in the Kyrgyz Republic, highlighting regional disparities and the critical role of early detection.

Methods: A retrospective data analysis was conducted on 1,338 women diagnosed with cervical cancer from 2012 to 2017 in the Kyrgyz Republic. Data were sourced from national health centers, focusing on sociodemographic metrics, clinical staging, and regional distributions. The study utilized statistical analysis to evaluate prevalence and mortality rates, employing the analysis of variance for comparison, significance, and analyzing trends over time.

Results: The prevalence of cervical cancer in the Kyrgyz Republic increased from 97.5 per 100,000 females in 2012 to 105.3 per 100,000 in 2017, with mortality rates of 8.3-9.7%. Notably, regional disparities were evident, with Chui, Osh, Jalal-Abad, and Bishkek experiencing increased prevalence rates, while Talas, Issyk-Kul, Naryn, and Batken reported decreased prevalence. Screening programs, particularly the introduction of Pap smears, have been effective in reducing both prevalence and mortality rates in areas with broad population coverage. However, the study highlighted significant variations in outcomes across different regions, underscoring the importance of targeted prevention and screening efforts.

Conclusions: The study confirms the ongoing challenge of cervical cancer in the Kyrgyz Republic, emphasizing the need for improved screening and prevention strategies to address disparities in outcomes. The introduction of pilot screening programs represents a crucial step forward. However, the findings also point to the necessity for enhanced oncological literacy among primary care physicians and the implementation of comprehensive strategies to overcome socioeconomic and regional barriers to effective cervical cancer prevention and treatment. The reduction in prevalence observed in 2016 suggests progress, highlighting the potential impact of focused prevention and screening initiatives.

## Introduction

Cervical cancer is a major health issue for women, ranking as the fourth most common type of cancer and posing a significant global health concern [1-3]. Approximately 604,000 new cases of cervical cancer are reported each year, with 342,000 female deaths globally attributed to this condition [2]. The primary cause of cervical cancer is a persistent infection caused by high-risk forms of human papillomavirus (HPV) [4]. This disease accounts for a significant proportion of female genital organ cancer cases in certain nations. Fortunately, effective preventative measures, such as screening and immunization programs, are available [5].

Despite recent advances in the prevention, screening, diagnosis, and treatment of cervical cancer, notable disparities still exist in these aspects at both the regional and global levels [6]. Racial and ethnic differences in this condition are largely attributed to variations in screening programs and socioeconomic status [7]. However, cervical cancer remains prevalent in certain regions, such as Asian nations, where access to screening programs is limited [8]. Cervical cancer screening aims to detect anomalies during the early stages of cancer development.

Despite the implementation of cervical cancer screening programs, the prevalence and mortality rates have continued to rise [2,9]. Compelling epidemiological evidence suggests that the introduction of Pap smears in countries with well-established screening programs and broad population coverage has led to a significant decrease in both the incidence and mortality rates of this condition [10,11].

The prevalence and mortality rates for cervical cancer are high in several countries located in Eastern Europe and Central Asia, except those within the European Union [1,12]. The Kyrgyz Republic, Kazakhstan, Moldova, Russia, and Ukraine report age-standardized incidence rates exceeding 14.0 cases per 100,000, surpassing those observed in the European Union [1,13].

Early detection is critical for preventing the progression from benign to malignant cervical conditions. This study aimed to investigate and analyze the prevalence and progression of cervical cancer in the Kyrgyz Republic between 2012 and 2017 and examine the patterns of prevalence and mortality associated with cervical cancer among women in different regions of the Kyrgyz Republic.

## Materials and methods

This is a retrospective data analysis of 1,338 women with cervical cancer, with age groups ranging from 0 to 65 years and >65 years. The data used for the study were collected from the Health Care Center, National Center of Oncology and Hematology of the Kyrgyz Republic between 2012 and 2017.

The inclusion criteria were as follows: women who received treatment for various types of cervical cancer, including squamous cell carcinoma, adenocarcinoma, or other undefined metastatic, persistent, or recurrent diseases, and had a follow-up regimen. The researchers ensured confidentiality by maintaining the privacy of the data collected from patients who provided informed consent. This study was conducted in accordance with the ethical standards outlined in the Declaration of Helsinki and approved by the Institutional Bioethics Committee of Kyrgyz Russian Slavic University, Kyrgyz Republic (Protocol No. 5, dated March 20, 2023).

In this study, we observed and recorded the following characteristics and variables: sociodemographic metrics (prevalence, mortality, International Federation of Gynecology and Obstetrics clinical staging for cervical cancer), and the different regions of the Kyrgyz Republic (Chui, Talas, Osh, Issyk-Kul, Jalal-Abad, Naryn, Batken, and Bishkek).

The research process involved calculating the relative values (intensive, extensive, and credible) of the data. Data are presented as absolute frequencies (n), percentages (%), and means±standard deviations (M±SD). The significance of group differences was determined using the analysis of variance, with p-values used as the accuracy measure (p <0.05, p <0.01, and p <0.001, respectively). Further, this study calculated the time series (absolute growth and growth rate), rate of lost years of potential life, and economic losses associated with cervical cancer.

## Results

The prevalence of cervical cancer in the Kyrgyz Republic increased from 97.5±1.8 per 100,000 females in 2012 to 105.3±1.8 in 2017, which was statistically significant (p <0.001). In Bishkek, the number of cases increased from 80.7±4.1 in 2012 to 86.8±4.1 in 2015 and then decreased slightly in 2016 and 2017 (84.0±4.0 and 80.5±3.9, respectively; p >0.05). Moreover, the prevalence rate of cervical cancer in women in Osh increased by 1.4 times from 101.2±8.6 in 2012 to 145.1±9.9 in 2017, which was statistically significant (p <0.001). In the Batken region, the prevalence rate increased by a factor of 1.3 from 58.6±5.1 in 2012 to 77.3±5.5 in 2017, excluding 2013 (57.3±5.0), with a p = 0.01. Additionally, the prevalence rate in the Jalal-Abad region decreased from 2012 (69.4±3.6) to 2016 (56.2±3.1) and then slightly increased in 2017 (59.3±3.1), with statistical significance (p <0.05). Women in the Issyk-Kul area experienced a similar trend, with a 1.1-fold decrease from 2012 (165.9±8.5) to 2016 (149.1±7.9), followed by a slight increase in 2017 (150.6±7.9) (p >0.05). Furthermore, in the Osh and Naryn regions, the prevalence rate of cervical cancer increased by 1.4 and 1.1 times, respectively, from 2012 to 2017 (p >0.05). The prevalence rate in the Talas region increased by 1.2 times from 2012 (110.2±9.6) to 2016 (133.2±10.2) and then decreased slightly in 2017 (125.8±9.9) (p >0.05). Finally, in the Chui region, the prevalence rate fluctuated between periods of increase and decrease, without significance (p >0.05) (Table 1).

**Table 1:** Prevalence of cervical cancer by regions of the Kyrgyz Republic from 2012 to 2017 Data indicate the prevalence rate per 100,000 population mean (M)±standard deviation (SD) in each studied group. *p <0.05, **p <0.01, ***p <0.001.

S. no.	Regions	2012	2013	2014	2015	2016	2017
		M±SD	M±SD	M±SD	M±SD	M±SD	M±SD
1.	Chui	159.5±6.8	155.0±6.8	159.5±6.8	161.8±6.9	159.1±6.8	164.1±6.9
2.	Talas	110.2±9.6	112.6±9.6	121.7±9.7	127.9±9.8	133.2±10.2	125.8±9.9
3.	Osh	63.0±5.4	70.7±5.5	78.2±5.6	81.2±5.7	83.8±5.8	89.4±6.0
4.	Issyk-Kul	165.9±8.5	156.3±7.8	154.2±7.6	152.4±7.8	149.1±7.9	150.6±7.9
5.	Jalal-Abad	69.4±3.6	67.3±3.5	60.3±3.3	57.5±3.1	56.2±3.1	59.3±3.1*
6.	Naryn	147.0±7.5	147.6±7.5	159.2±7.8	160.3±7.8	168.7±7.9	170.0±7.9
7.	Batken	58.6±5.1	57.3±5.0	61.6±5.1	65.7±5.2	70.7±5.3	77.3±5.5
8.	Osh	101.2±8.6	107.7±8.7	111.6±8.1	124.5±8.1	135.9±8.3	145.1±9.9***
9.	Bishkek	80.7±4.1	83.1±3.6	85.1±3.7	86.8±4.1	84.0±4.0*	80.5±3.9*
10.	Kyrgyz Republic	97.5±1.8	97.8±1.8	100.3±1.9	102.0±2.0	102.7±2.0	105.3±1.8***

The Kyrgyz Republic exhibited a high prevalence of stages I-II cervical cancer during the years 2012 to 2015, with the highest rates recorded in 2012, 2014, and 2015. The prevalence decreased in 2016 compared to that in 2013 but exhibited an uptick in 2017. Throughout the years, no significant differences in prevalence were observed. Stage III cervical cancer was second in prevalence throughout the study period, experiencing a marginal decline except in 2014 when it reached 93.4±12.0. The prevalence for 2017 marginally increased to 79.4±10.7 from 67.2±8.4 in 2016, without significance. In 2016, the prevalence of stage IV cervical cancer was at its highest level at 42.9±8.0 in contrast to its lowest prevalence in 2015 at 25.0±4.7, also without significance (Table 2).

**Table 2:** Prevalence of cervical cancer by stages and regions of the Kyrgyz Republic Data indicate the prevalence rate per 100,000 population (n), mean (M)±standard deviation (SD) in each studied group. *p <0.05, **p <0.01, ***p <0.001.

S. no.	Regions	Stages	2012		2013		2014		2015		2016		2017	
			n	M±SD	n	M±SD	n	M±SD	n	M±SD	n	M±SD	n	M±SD
1.	Chui	I-II	51	29.6±4.1	37	21.6±3.0	76	36.2±4.1*	70	32.7±3.6*	64	31.4±3.7*	61	29.5±3.4
		III	35	18.7±2.4	17	9.2±1.2	39	22.2±3.0	35	19.8±2.7	24	12.9±6.8	30	17.6±2.5
		IV	8	6.9±1.4	6	4.7±0.9***	17	12.1±2.1	10	7.8±1.5	15	11.7±2.2	10	6.9±1.1
2.	Talas	I-II	14	8.1±1.6	7	4.0±0.5	12	5.7±0.6	17	7.9±0.9	12	5.3±0.7	11	5.3±0.6
		III	3	1.6±0.2	12	6.5±0.8	11	6.3±0.9	7	3.9±0.5	8	4.3±0.5	9	5.3±0.7
		IV	-	-	-	-	-	-	-	-	1	0.8±0.1	-	-
3.	Osh	I-II	40	23.2±3.4	36	21.0±3.0	28	13.3±1.5	31	14.5±1.6	30	14.7±1.7	49	23.7±2.8
		III	35	18.7±2.4	40	21.7±2.8	39	22.2±3.0	36	20.9±2.8	37	19.9±2.6	28	16.4±2.3
		IV	7	6.0±1.3	5	4.0±0.8***	9	6.4±1.1	8	6.3±1.2	9	7.0±1.3	3	2.0±0.3***
4.	Issyk-Kul	I-II	24	13.9±2.0	34	19.9±2.8	36	17.1±2.0	29	13.5±1.5	23	11.3±1.3	29	14.0±1.6
		III	16	8.5±1.1	25	13.5±1.8	16	9.1±1.3	18	10.2±1.4	12	6.4±0.8	15	8.8±1.2
		IV	7	6.0±1.3	5	4.0±0.8	4	2.8±0.5	7	5.5±1.0	4	3.1±0.6	4	1.9±0.2
5.	Jalal-Abad	I-II	40	23.2±3.3	24	14.0±2.0	49	23.4±2.7	46	21.5±2.4	43	21.0±2.7	40	19.6±2.3
		III	41	21.9±2.8	25	13.5±1.8	23	13.1±1.8	17	9.6±1.3	9	7.0±0.9***	24	14.1±2.0
		IV	7	6.0±1.3	8	6.4±1.3	15	10.7±1.9	15	11.7±2.2	16	12.5±2.4	17	11.7±2.0
6.	Naryn	I-II	10	5.8±0.8	14	8.2±1.1	26	12.4±1.7	18	8.4±1.0	19	9.3±1.1	16	7.7±0.9
		III	12	6.4±0.8	10	5.4±0.7	9	5.1±0.7	8	4.5±0.6	8	4.3±0.5	8	4.7±0.7
		IV	2	1.7±0.3	1	0.8±0.1	4	2.8±0.5	1	0.8±0.1	1	0.8±0.1	8	5.5±0.9
7.	Batken	I-II	7	4.0±0.5	16	9.3±1.3	18	8.6±1.0	21	9.8±1.3	22	10.8±1.3	19	9.1±1.0
		III	6	3.2±1.7	13	17.0±0.9	8	4.5±0.6	11	6.2±0.8	10	5.4±0.6	8	4.7±0.7
		IV	1	0.9±0.1	2	1.6±0.3	-	-	3	2.3±0.4	2	1.5±0.3	7	4.8±0.8
8.	Osh	I-II	21	12.1±1.7	15	8.7±1.1	10	4.7±0.5	15	7.0±0.1	13	6.4±0.7	20	9.7±1.1
		III	11	5.9±0.8	11	6.0±0.8	10	5.7±0.8	15	8.5±1.1	12	6.4±0.8	6	3.5±0.5
		IV	2	1.7±0.3	7	5.6±1.1***	1	0.7±0.1***	6	4.7±0.9	6	4.6±0.9	3	2.0±0.3***
9.	Bishkek	I-II	56	32.5±4.5	52	10.4±4.3	60	28.6±3.3	73	34.1±3.8	42	20.6±2.4	48	23.2±2.7
		III	10	5.3±0.7	11	5.9±0.8	9	5.1±0.7	9	5.1±0.7	5	2.7±0.9	7	4.1±0.6
		IV	4	3.4±0.4***	3	2.4±0.4	2	1.4±0.2	2	1.6±0.3	1	0.8±0.1***	-	-
10.	Kyrgyz Republic	I-II	259	150.4±18.5	235	137.5±17.3	315	150.3±15.3	320	149.5±14.8	268	131.4±14.0	293	141.1±14.6
		III	167	89.2±10.8	164	88.8±11.0	164	93.4±12.0	156	88.6±11.4	125	67.2±8.4	135	79.4±10.7
		IV	45	38.8±8.0	37	29.5±5.7	53	37.8±6.9	52	25.0±4.7	55	42.9±8.0	52	36.0±6.0

The highest illness rates in the Republic are found in the Chui, Osh, and Jalal-Abad regions, as well as in the cities of Bishkek and Osh. In the Chui area, the prevalence of stages I-II cervical cancer increased significantly in 2014 (36.2±4.1), 2015 (32.7±3.6), and 2016 (31.4±3.7), with p <0.05. However, the prevalence of stage III cervical cancer varied throughout the study period, including a decrease of more than twofold to 9.2±1.2 in 2013 (Table 2).

The prevalence of stage IV was similar, showing a significant increase from 12.1±2.1 in 2014 to 4.7±0.9 in 2013 (p <0.001). The Osh area reported the highest prevalence of cervical cancer in stages I-II in 2012 (23.2±3.4), 2013 (21.0±3.0), and 2017 (23.7±2.8), without statistical significance (p >0.05).

Stage III was the most commonly reported cervical cancer from 2013 to 2015, without significance (p >0.05). The prevalence of stage IV varied from 6.0±1.3 in 2012 to 7.0±1.3 in 2016, but was not statistically significant (p >0.05); however, the difference in the prevalence of stage IV cervical cancer for 2013 and 2017 was statistically significant with rates of 4.0±0.8 and 2.0±0.3, respectively (p <0.001).

The prevalence of cervical cancer in the Jalal-Abad region fluctuated between 2010 and 2019, including a decrease to 14.0±2.0 in 2013. Furthermore, the prevalence of stage III cervical cancer varied, with a low point in 2016 at 7.0±0.9, which was statistically significant (p <0.001), and another low point in 2017 at 14.1±2.0, which was not significant (p >0.05). However, the prevalence of stage IV cervical cancer was consistently high but did not prove significant for 2015, 2016, and 2017 (p >0.05).

The prevalence of cervical cancer in Bishkek, the Kyrgyz Republic, was highest in 2015 with 34.1±3.8 cases reported, followed by 2012 and 2014 with 32.5±4.5 and 28.6±3.3 cases, respectively. Differences in cervical cancer prevalence between these years were not significant (p >0.05). In 2016 and 2017, the prevalence was high at 20.6±2.4 and 23.2±2.7 cases, respectively, with no significant difference (p >0.05). Furthermore, in 2013, the prevalence was low at 10.4±4.3 cases. The prevalence of stage III cervical cancer remained relatively stable from 2012 to 2015, with values of 5.3±0.7, 5.9±0.8, and 5.1±0.7 cases, respectively (p >0.05). In 2016, the prevalence decreased to 2.7±0.9 cases and increased to 4.1±0.6 cases in 2017, with no significant difference (p >0.05). Finally, the prevalence of stage IV cervical cancer significantly decreased from 3.4±0.4 cases in 2012 to 0.8±0.1 cases in 2016 (p <0.001), with no cases reported in 2017.

The highest prevalence of stages I-II cervical cancer was observed in Bishkek in 2014 and 2015, with no significant difference between these years (p >0.05). Moreover, between 2012 and 2017, the prevalence rate of stage III cervical cancer remained stable with no significant change (p >0.05). In 2016, the prevalence of stage IV cervical cancer significantly decreased in Bishkek (p <0.001). In Osh, the prevalence rate of stages I-II cervical cancer peaked in 2012, recording 12.1±1.7 cases, while the proportion of patients diagnosed with stage III was highest in 2015 at 8.5±1.1. The number of patients with stage IV surged in 2013 to 5.6±1.1, but significantly diminished in 2014 (0.7±0.1) and again in 2017 (2.0±0.3), evidencing a substantial difference across all years (p <0.001).

The prevalence of cervical cancer was found to be minimal in the areas of Talas, Issyk-Kul, Naryn, and Batken, with the exception of stages I-II cases in the Issyk-Kul region. Remarkably, no instances of stage IV disease were documented in the Talas and Batken regions, except for the years 2016 (0.8±0.1) and 2014, respectively.

In 2017, the primary prevalence rate increased by 75.0% for those aged 20-24 years and 83.3% for those aged 25-29 years in 2013, followed by a 31.8% increase in 2014 and a 20.6% increase in 2015 (p <0.001) (Table 3).

**Table 3:** Dynamics of newly diagnosed patients with cervical cancer in the Kyrgyz Republic from 2012 to 2017 Data indicate the prevalence rate per 100,000 population (n) and growth rate (GR). *p <0.05, **p <0.01, ***p <0.001.

S. no.	Age (years)	Years											
		2012		2013		2014		2015		2016		2017	
		n	GR	n	GR	n	GR	n	GR	n	GR	n	GR
1.	0-6	0	-	0.2	0	0	0	0	0	0	0	0	0
2.	7-14	0	-	0	0	0	0	0	0	0	0	0.2	0
3.	15-19	0	-	0	0	0	0	0.4	0	0	0	0	0
4.	20-24	0	-	1.0	0	0	-100.0	1.1	0	0.4	-63.6	0.7	+75.0***
5.	25-29	1.2	-	2.2	+83.3***	2.9	+31.8***	3.5	+20.6***	1.7	-51.4	1.7	0
6.	30-34	7.6	-	9.8	+28.9	11.2	+14.2	6.7	-40.1	8.0	+19.4	6.8	-15.0
7.	35-39	32.4	-	25.8	-20.3	19.8	-23.2	17.8	-10.1	16.9	-5.0	25.1	+48.5
8.	40-44	41.2	-	32.0	-22.3	30.7	-4.0	43.4	+41.3	31.4	-27.6	37.5	+19.4
9.	45-49	54.8	-	42.6	-22.2	54.3	+27.4	61.9	+13.9	50.5	-18.4	38.2	-24.3
10.	50-54	51.5	-	58.1	+12.8	69.6	+19.7	61.6	-11.4	57.2	-7.1	64.7	+13.1
11.	55-59	63.0	-	52.7	-16.3	70.3	+33.3	60.2	-14.3	52.4	-12.9	57.9	+10.4
12.	60-64	48.2	-	51.6	+7.0	75.7	+46.7	60.5	-20.0	48.5	-19.8	37.5	-22.6
13.	65 and older	42.6	-	37.8	-11.2	52.7	+39.4	43.1	-18.2	35.4	-17.8	37.5	+5.9
	Total	16.6	-	15.2	-8.4	18.1	+19.0	17.6	-2.7	14.8	-15.9	15.5	+4.7

For those aged 30-39 years, the prevalence of cervical cancer increased by 28.9%, 14.2%, 19.4%, and 48.5% in 2013, 2014, 2016, and 2017, respectively. For those in the 40-44 year age group, prevalence increased by 41.3% in 2015. In the 45-49 year age group, an increasing trend of 27.4% and 13.9% was observed in 2014 and 2015, respectively. For females aged 50-54 years, an increase of 12.8%, 19.7%, and 13.1% was observed in 2014, 2013, and 2017, respectively. Moreover, in the 55-59 year age range, the prevalence of cervical cancer increased by 35.3% in 2014 and 10.4% in both 2013 and 2017. In 2013 and 2014, the number of patients newly diagnosed with cervical cancer increased by 7.0% and 46.7%, respectively. For those aged ≥ 65 years, the prevalence of cervical cancer diagnoses increased by 39.4% in 2014 and 5.9% in 2017.

Evaluating cancer mortality rates is essential to determine the effectiveness of current cancer control measures. A significant increase in mortality was observed in the age groups 25-29 and 30-34 years in 2016, with 1.2% and 2.3%, respectively, and 0.9% and 2.8% in 2013, respectively (p <0.001). Additionally, for those aged 35-39 years and 40-44 years, mortality increased by 6.9% and 10.8% in 2015, respectively, (p <0.001). Furthermore, in 2013, the mortality rate associated with cervical cancer increased by 15.3% and 20.0% in the age groups 45-49 years and 50-54 years, respectively, and by 17.0% and 18.5% in 2015, respectively. Among those aged 55-59 years, the mortality rate increased by 9.3% in 2014 and by 14.8% in 2015 (p <0.001). The mortality rates for those aged 60-64 years increased by 14.4% in 2016 and 12.0% in 2014, while for those aged ≥ 65 years, the mortality rates increased by 27.8% in 2017 and 24.4% in 2014 (Table 4).

**Table 4:** Age dynamics of mortality from cervical cancer in the Kyrgyz Republic from 2012 to 2017 Data indicate the number of deaths (n) and percentages (%). *p <0.05, **p <0.01, ***p <0.001.

S. no.	Age (years)	2012	2013	2014	2015	2016	2017
		n (%)	n (%)	n (%)	n (%)	n (%)	n (%)
1.	0-4	-	-	1 (0.5)	-	-	-
2.	5-14	-	-	-	-	-	-
3.	15-19	-	-	-	-	-	-
4.	20-24	1 (0.5)	1 (0.5)	-	1 (0.4)	-	-
5.	25-29	1 (0.5)	2 (0.9)***	2 (0.9)	1 (0.4)	3 (1.2)	1 (0.4)
6.	30-34	3 (1.5)	6 (2.8)***	4 (1.9)	10 (3.9)	5 (2.3)	7 (3.1)
7.	35-39	18 (8.7)	18 (8.3)	13 (6.2)	18 (6.9)	16 (7.2)	7 (3.1)
8.	40-44	27 (13.1)	25 (11.6)	15 (7.2)	28 (10.8)***	23 (10.4)	23 (10.1)
9.	45-49	29 (14.1)	33 (15.3)	30 (14.4)	44 (17.0)	37 (16.7)	29 (12.8)
10.	50-54	32 (15.5)	43 (20.0)	37 (17.7)	48 (18.5)	28 (12.6)	41 (18.1)
11.	55-59	20 (9.7)	20 (9.3)***	31 (14.8)***	38 (14.7)	33 (14.9)	27 (11.9)
12.	60-64	23 (11.1)	19 (8.8)	25 (12.0)	23 (8.8)	32 (14.4)	29 (12.8)
13.	65 and older	52 (25.2)	48 (22.3)	51 (24.4)	48 (18.5)	45 (20.2)	63 (27.8)
14.	Total	206 (7.1)	215 (7.4)	209 (7.2)	259 (8.9)	222 (7.6)	227 (7.8)

## Discussion

Cervical cancer poses a significant challenge to global health, and improvement in prevention and control measures is needed [10,14]; importantly, numerous studies from diverse countries have documented a persistent increase in its prevalence [12-15].

In numerous countries with substantial populations, including China (10.7 cases per 100,000 women-years), India (18.0 cases per 100,000 women-years), Indonesia (24.4 cases per 100,000 women-years), Russia (14.1 cases per 100,000 women-years), and Brazil (12.7 cases per 100,000 women-years), the incidence of cervical cancer surpasses the threshold established by the World Health Organization [15].

In this study, the prevalence of cervical cancer in the Kyrgyz Republic increased from 97.5±1.8 per 100,000 women in 2012 to 105.3±1.8 in 2017 (p <0.001). The Kyrgyz Republic has a high age-standardized rate of more than 14.0 cases per 100,000, which exceeds that of the European Union [1,13].

The mortality rate of cervical cancer among patients in the Kyrgyz Republic varied between 8.3% and 9.7% throughout the study. Furthermore, several Central Asian nations, including Kazakhstan, Moldova, Turkmenistan, and the Kyrgyz Republic, experienced an increase in age-standardized mortality rates for cervical cancer of more than 7.0 cases per 100,000 people, surpassing that of the European Union [1,13].

The National Control and Prevention Strategy for Oncological Diseases in the Kyrgyz Republic for 2021-2025 seeks to implement a pilot mammography screening program for women aged 35-49 years and initiate a pilot screening for cervical cancer for individuals aged 30-49 years employing techniques such as visual inspection with acetic acid and the Pap and HPV tests. Currently, a pilot community-based cervical cancer screening study using visual inspection with acetic acid is being conducted in the Sokuluk region, which represents approximately 3% of the population of the Kyrgyz Republic [16].

In this research, a notable decrease in cervical cancer prevalence was observed in the Kyrgyz Republic in 2016, representing a 12.1% reduction in stages I-II cancer and a 21.4% decrease in stage III cancer compared to data from 2015 and 2013, respectively. Despite the increase in cervical cancer cases in many countries, including the Kyrgyz Republic, before 2013, the implementation of a screening program resulted in a decline in incidence [17,18]. In 2013, a cytological screening program was initiated; however, no explicit information is available on the specific age categories targeted by the government program.

The relationship between cervical cancer and a country's level of development and population is exemplified by the challenges faced by many developing nations. A clear link between cervical cancer and the geographical location of a country, contributing to the disparities observed between regions, has been established.

Furthermore, precancerous lesions, which are more common in industrialized nations, play a significant role in these disparities. Enhancing screening coverage, administration, and quality control of screening programs and providing feedback to participants to address these issues is crucial. In particular, defining the economic loss associated with the death of females with genital malignancies to effectively utilize health resources is essential; this information can be used to justify the need for cancer care in the population [19].

This study intended to address a significant gap in healthcare research on patient participation in cervical cancer screening by combining administrative and comprehensive survey data. This study has some limitations, particularly those stemming from the qualitative nature of the data. Cervical cancer diagnosis may be affected by associated risk factors; however, we did not consider these factors in our study.

## Conclusions

In the Kyrgyz Republic, the prevalence rate of cervical cancer increased from 97.5 per 100,000 individuals in 2012 to 105.3 per 100,000 individuals in 2017, which was statistically significant (p <0.001). This increase was observed in all regions, including Chui, Osh, Jalal-Abad, and Bishkek. One death was detected in the age group of 0-4 years in 2014. In 2017, the total lost years of potential life due to cervical cancer was 13.47 years. Primary care physicians and their insufficient oncological literacy are the primary causes of a significant proportion of neglected cancers.
